# Characterization of the autophosphorylation property of HflX, a ribosome‐binding GTPase from *Escherichia coli*

**DOI:** 10.1002/2211-5463.12065

**Published:** 2016-06-08

**Authors:** Aditi Ghosh, Dipak Dutta, Kaustav Bandyopadhyay, Pradeep Parrack

**Affiliations:** ^1^ Department of BiochemistryBose InstituteKolkataIndia; ^2^ Present address: CSIR‐Institute of Microbial TechnologySector 39AChandigarh160036India; ^3^ Present address: Department of Plant BiologyThe Samuel Roberts Noble FoundationArdmoreOKUSA

**Keywords:** autophosphorylation, HflX(C), TRAFAC‐GTPase

## Abstract

*Escherichia coli* HflX belongs to the widely distributed but poorly characterized HflX family of translation factor‐related GTPases that is conserved from bacteria to humans. A 426‐residue polypeptide that binds 50S ribosomes and has both GTPase and ATPase activities, HflX also exhibits autophosphorylation activity. We show that HflX(C), a C‐terminal fragment of HflX, has an enhanced autophosphorylation activity compared to the full‐length protein. Using a chemical stability assay and thin layer chromatography, we have determined that phosphorylation occurs at a serine residue. Each of the nine serine residues of HflX(C) was mutated to alanine. It was found that all but S211A retained autophosphorylation activity, suggesting that S211, located in the P‐loop, was the likely site for autophosphorylation. While the S211A mutant lacked the autophosphorylation site, it possessed strong GTP binding and GTPase activities.

**Abbreviations**
HflX(C): HflX C‐terminal domain
HflX(N): HflX N‐terminal domain
TRAFAC‐GTPases: translation factor‐related guanosine triphosphatases

The HflX family of translation factor‐related guanosine triphosphatases (TRAFAC‐GTPases), despite being widely distributed across species, is a poorly characterized protein family 1, 2. The *E*. *coli* HflX is a 426‐residue polypeptide (MW, 48.3 kDa) that has four GTP‐binding motifs, including the nucleotide‐binding motifs of P‐loop GTPases 3. *E. coli* HflX hydrolyses not only GTP but also ATP, liberating inorganic phosphate 4. The broad phylogenic distribution pattern of HflX GTPases in bacteria, archaea, and eukaryotes, including humans 5, suggests a basic cellular function for this protein family 6. HflX has also been reported to interact with the 50S ribosomal subunit in the presence of any of the purine nucleotides GTP, GDP, ATP, or ADP 7. While *E. coli* HflX binds and hydrolyses both ATP and GTP, only the GTP‐hydrolysis activity is stimulated by 50S ribosome binding 7.

The three‐dimensional structure of *E. coli* HflX is unknown, but the X‐ray crystallographic structure of HflX from *Sulfolobus solfataricus* has been reported 8. This structure displays a two‐domain architecture with a novel ‘HflX domain’ at the N‐terminus and a classical ‘G‐domain’ at the C‐terminus. The cleft between the two domains accommodates the nucleotide‐binding site. From sequence alignment it has been found that the N‐terminal domain of *E. coli* HflX has high similarity with its Sulfolobus counterpart, but the C‐terminal domain is dissimilar 8.

HflX is part of a large polycistronic operon, genes of which are regulated by multiple σ70‐ and σ32‐dependent promoters and encode proteins of diverse functions. It has been shown that HflX splits ribosomes when overexpressed 9. Since *hfl*X is upregulated upon heat shock 10, it may act as a regulator of translation by splitting ribosomes under conditions of stress. Previously we showed that deletion of *E. coli hfl*X causes the bacterial cells to be extremely sensitive to manganese, characterized by arrested cell growth, filamentation, slower rate of replication, and DNA damage 11.

Several P‐loop bacterial proteins exhibit autophosphorylation activity, as exemplified by Era from *E. coli* 12, Obg from *B. subtilis* 13, or CgtA from *C. crescentus* 14. The P‐loop protein HflX also exhibits autophosphorylation activity in the presence of GTP but not in the presence of ATP 11. It was proposed that manganese‐mediated autophosphorylation of HflX played an important role in manganese, zinc and iron homeostasis in *E. coli* cells 11. Moreover, different metal ions induced degrees of phosphorylation in the following order: Mn^2+^ > Mg^2+^ > Ca^2+^ 11. GTP‐dependent autophosphorylation of HflX using Mn as a primary cofactor was useful in demonstrating the relationship between HflX and Mn homeostasis.

More recently, it has been shown that the N‐terminal glycine‐rich domain of HflX has a Rossman‐like noncanonical NTPase motif, aside from the C‐terminal canonical P‐loop NTPase nucleotide‐binding motif (G‐domain) 15. Interestingly, these nucleotide‐binding domains of HflX influence each other's NTPase activity 15. To check the individual autophosphorylation properties of the domains, we cloned, overexpressed, and purified the N‐terminal and C‐terminal domains of HflX. While the C‐terminal domain (HflX(C)) showed a strong autophosphorylation activity, no such activity could be detected for the N‐terminal domain (HflX(N)). Further, unlike full‐length HflX, HflX(C) could use both Mn^2+^ and Mg^2+^ cofactors equally well for autophosphorylation. We also examined the nature of the phosphoamino acid by chemical stability assay and show that the autophosphorylation involves a serine residue of HflX, and identified Ser211 as the site of autophosphorylation.

## Materials and methods

### Materials

#### Chemicals

Various chemicals and reagents were obtained from E. Merck (Mumbai, India) Limited; Sisco Research Laboratories Pvt. Ltd., Mumbai, India; HiMedia Laboratories Limited (Mumbai, India) and from Sigma Chemical Co., St. Louis, MO, USA. The Ni^+2^‐nitrilotriacetic acid resin (QIAGEN GmbH, Hilden, Germany), dNTP mix (Fermentas Life Sciences, Waltham, MA, USA), Immobilon‐P (PVDF, polyvinylidene fluoride) membrane (Millipore, Billerica, MA, USA) and [γ^32^‐P] GTP (specific activity 4500 Ci·mmol^−1^) (BRIT, Mumbai, India) were purchased from the indicated sources.

Cellulose thin layer (100 μm) chromatography plates (polyester sigmacell type 100 cellulose) of dimensions 20 cm × 20 cm, were purchased from Sigma.

#### Enzymes

All enzymes used for the manipulation of DNA were obtained either from United States Biochemical Corporation (Clevelend, OH, USA) or from Promega Biosciences (Madison, WI, USA) or RocheApplied Sciences (Basel, Switzerland).

#### Bacterial strains and plasmids

The expression vectors (pET28a from Novagen, Madison, WI, USA) and *E. coli* strains XL1Blue, BL21 (DE3), Top10, and pETX (*hfl*X in pET28a) were used in this study, and were maintained at −70 °C as glycerol stocks. The plasmids pS176A, pS191A, and pS201A were obtained from D. Dutta, IMTECH, Chandigarh, India.

### Methods

#### Cloning, expression, and purification of HflX(C)

The *hflX(C)* gene was PCR‐amplified by the primers XCF and XCR (see Table S1 for sequence) using pETX as the template. The PCR product was digested with *Bam*HI and *Xho* I and ligated into the corresponding sites of the expression vector pET28a. The resulting plasmid pAG01 was transformed into BL21 (DE3) strain. Following an overnight induction at 16 °C with 0.4 mm IPTG, the expressed protein was purified through a Ni–nitrilotriacetic acid agarose column pre‐equilibrated with buffer A (Table S2). Elution was done by the addition of 500 mm of imidazole to buffer A. The protein obtained was 98% pure as estimated from a 13.5% SDS/PAGE followed by Coomassie staining.

#### Cloning, expression, and purification of HflX(N)

The *hflX(N)* gene was amplified by PCR using pETX as template with the XNF and XNR primers (Table S1). The PCR fragments containing the *hfl*X*(N)* gene were digested by *Bam*HI and *Hin*dIII and ligated into the corresponding sites of the expression vector pET28a. The resulting plasmid pAG02 was transformed into BL21 (DE3) strain. Following overnight induction at 16 °C with 0.4 mm IPTG, the expressed protein was retained in the pellet fraction. Therefore, the protein was purified in the denatured condition (using urea as denaturant) through a Ni–nitrilotriacetic acid agarose column pre‐equilibrated with lysis buffer (buffer L, Table S2). Following elution (by the addition of 500 mm imidazole to the above buffer), the protein was renatured by step dialysis using Buffer DU4, Buffer DU2, Buffer DU0, and Buffer DGE sequentially (Table S2) for stepwise removal of urea. The protein obtained was estimated from a 13.5% SDS/PAGE following Coomassie staining.

#### Autophosphorylation assay

Autophosphorylation assay was carried out by incubating 5 μm protein with 10 μCi [γ^32^‐P] GTP (or, ATP) (specific activity 4500 Ci·mmol^−1^) in 20 μL phosphorylation buffer P (Table S2) at 37 °C. The reaction was terminated by the addition of 1 mm of GTP and 5× SDS/PAGE sample‐loading buffer and applied to a 15% SDS/PAGE. After electrophoresis, the gel was stained with Coomassie blue to visualize the protein, dried and subjected to autoradiography.

#### GTP‐binding assay of mutant proteins

GTP‐binding assay was done following 16 and 4. About 10 μg of protein was incubated at 30 °C with 20 μL of buffer G1 (Table S2) containing 20 μCi of [α‐^32^P] GTP (specific activity 3000 Ci·mmol^−1^) and 10 μm of cold GTP for 30 min. The samples were kept on ice and subjected to UV cross‐linking by a UV crosslinker (Stratalinker; Stratagene, San Diego, CA, USA) for 15 min at 5 cm from the UV source. The samples were boiled with SDS and run in a 12.5% SDS/PAGE; the gels were dried and autoradiographed.

#### GTPase assay

For each condition, the reaction mixture was prepared by dissolving the substrate (GTP) in reaction buffer G2 (Table S2) and was incubated at 37 °C. The reaction started immediately upon addition of the enzyme (HflX(C) or S211A, 3 μm) to the reaction mixture. About 200‐μL aliquots of the final reaction mixture were removed and the reaction was stopped by adding 10 μL of perchloric acid (17 m). About 200 μL of malachite green was added to this, and the volume was made to 1 mL by adding double distilled water. Absorbance at 630 nm was recorded.

#### Mutation and cloning of *hflX(C)* gene

Site‐directed mutagenesis kit (Stratagene) was used according to the manufacturer's instruction for site‐directed mutagenesis of the *hflX(C)* gene carried in the pAG01 plasmids. The primers used for mutagenesis to introduce the required point mutation in *hflX(C)* gene are listed in Table S1. The sequences of the resulting plasmids were confirmed by sequencing.

#### Chemical stability assay of the phospholinked amino acids

The chemical stability of the phosphorylated amino acid(s) of HflX(C) was assayed according to 17. Equal amounts of His_6_‐HflX(C) (15 μm) were phosphorylated, separated by SDS/PAGE and blotted onto a PVDF membrane. Individual blots were excised from the membrane and incubated in (a) 50 mm Tris‐HCl (pH 7.5), (b) 3 m NaOH, (c) 1 m HCl, or (d) 0.8 m hydroxylamine at 42 °C. After incubation for 1 h, each of the membranes was washed with distilled water, dried in air and exposed to an X‐ray film.

#### Thin layer chromatography of digested phosphorylated protein

His_6_‐HflX(C) (15 μm) was phosphorylated with GTP as above, separated by SDS/PAGE, and blotted onto a PVDF membrane. The membrane strips containing the ^32^P‐labeled protein were cut into pieces of approx. 2 × 2 mm with a razor blade, and transferred to a screw‐cap glass tube. About 500 μL 6(N) HCl was added to the tube which was closed tightly by screwing the cap, vortexed, and placed in a sand bath at 110 °C for 60 min for hydrolysis. The reaction was stopped by adding a large amount of water. The protein was then lyophilized and resuspended in 10 μL of TLC running buffer T (Table S2). Phosphoserine, phosphothreonine, and phosphotyrosine (1 μL each) were also spotted on to the cellulose plate as controls and run with the TLC running buffer. The plate was dried and developed by 0.1% ninhydrin solution in acetone and exposed to an X‐ray plate for autoradiography.

## Results

### Autophosphorylation of HflX and its different fragments

**Figure 1:**
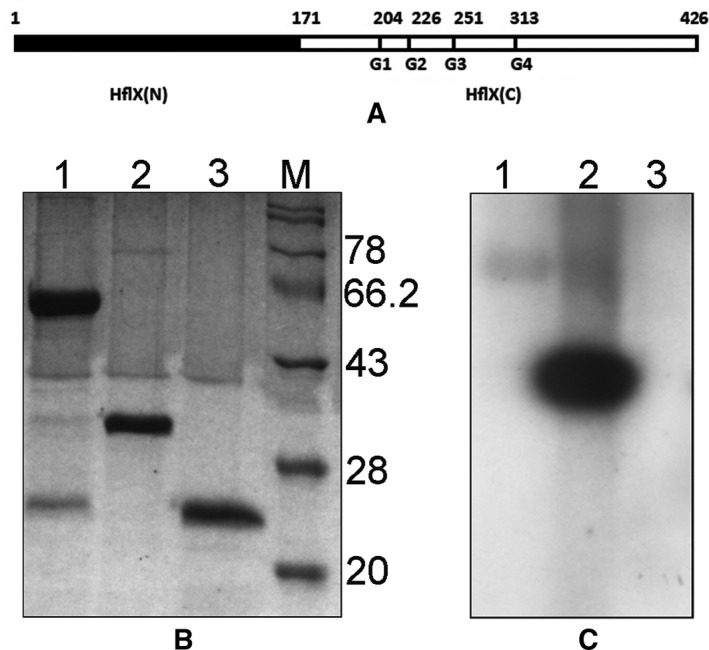
Autophosphorylation of HflX, HflX(C), and HflX(N). (A) A schematic representation of the primary sequences of the proteins used, along with the location of the G domains. (B) Autophosphorylation followed by 15% SDS/PAGE and staining by Coomassie Blue of his‐tagged HflX (lanes 1), HflX(C) (lanes 2), and HflX(N) (lanes 3). (C) An autoradiogram of the same gel. Lane M contains molecular weight markers, with molecular weights (KDa) indicated on the right.

**Figure 2:**
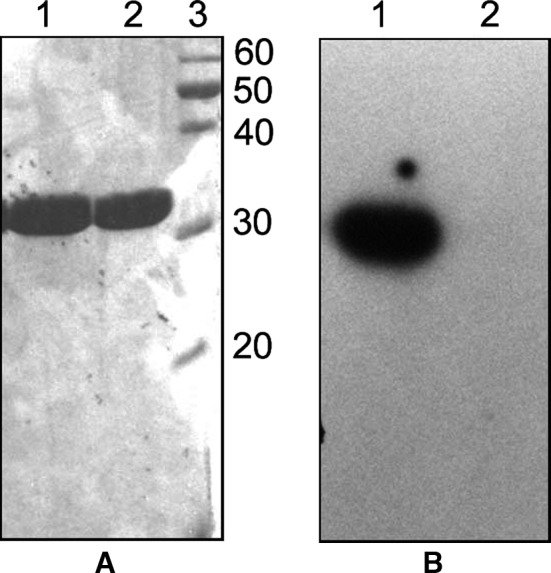
HflX(C) is not autophosphorylated in the presence of ATP. (A) Autophosphorylation of HflX(C) followed by 15% SDS/PAGE and staining by Coomassie Blue in the presence of GTP (Lanes 1) and ATP (Lanes 2). (B) An autoradiogram of the same gel. Lane 3 contains molecular weight markers, with molecular weights (KDa) indicated on the right.

During the early days of HflX purification, it was sometimes observed that the protein got fragmented during purification. Interestingly, the smaller fragment exhibited significant autophosphorylation activity (our unpublished results; described in Dutta 18). This observation led us to investigate the autophosphorylation activities of different regions of HflX, along with that of the full‐length protein. We chose to divide the protein into two parts: HflX(N) (residues 1–171, that lacked the G domains of HflX) and HflX(C) (residues 171–426, that contained all the four G domains of HflX). HflX(N) and HflX(C) were cloned and purified as N‐terminally (his)_6_‐tagged proteins as described under Materials and Methods. When equimolar concentrations of HflX, HflX(C), and HflX(N) were subjected to autophosphorylation assays, it was observed that the autophosphorylation signal of HflX(C) was nearly 5‐fold stronger compared to that for HflX. In contrast, HflX(N) showed no autophosphorylation (Fig. 1). In separate experiments, autophosphorylation of HflX and HflX(C) were examined after removal of the (his)_6_‐tags by thrombin, and similar results were obtained (not shown). Consequently, (his)_6_‐HflX(C) [referred simply as HflX(C)] was used in subsequent experiments carried out to characterize autophosphorylation. Like full‐length HflX, HflX(C) also is not autophosphorylated in the presence of ATP (Fig. 2).

#### HflX(C) is a GTP‐binding protein and a GTPase

**Figure 3:**
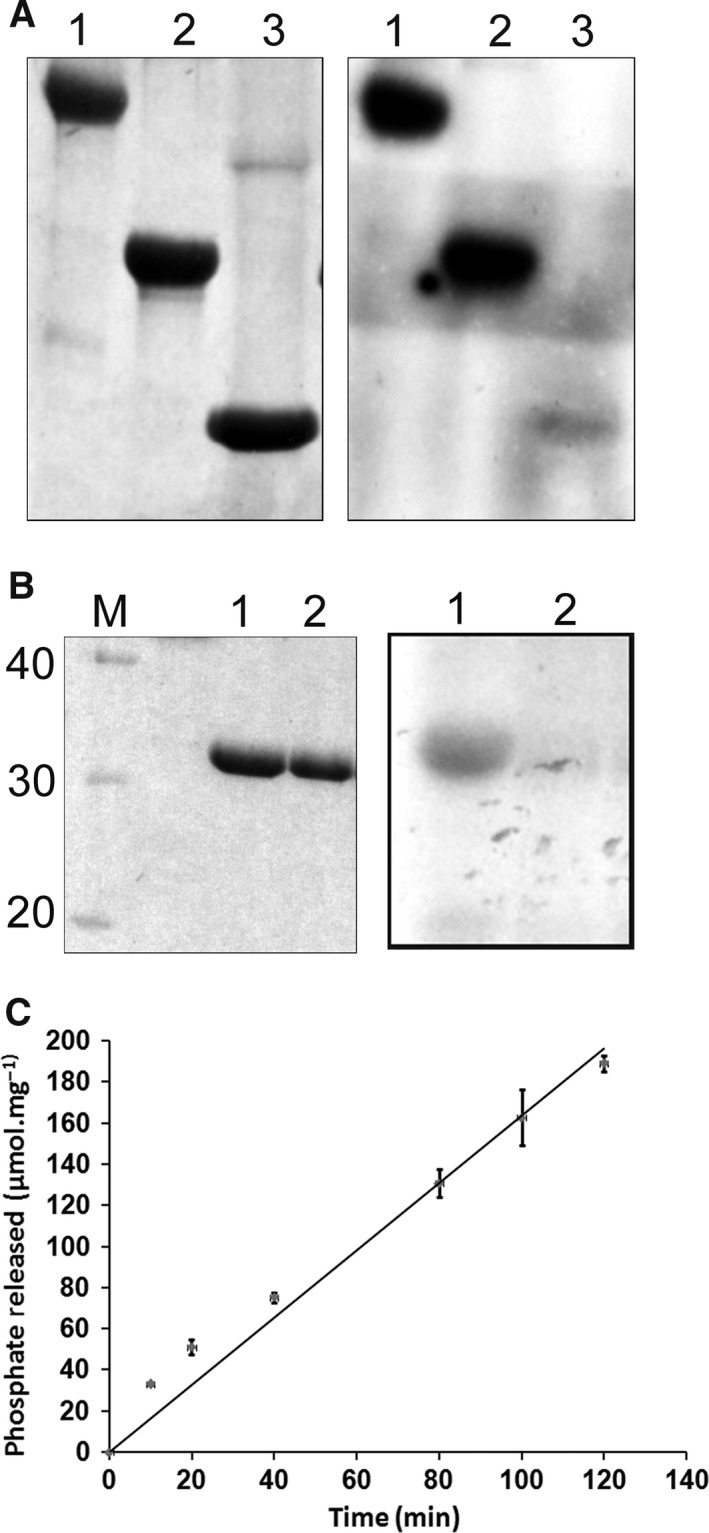
GTP‐binding and GTPase activity of HflX(C). (A) Equal concentrations of HflX (lanes 1), HflX(C) (lanes 2), and HflX(N) (lanes 3) were treated with [α‐^32^P] GTP followed by UV crosslinking and 15% SDS/PAGE. The gel was stained by Coomassie Blue (left panel) and also subjected to autoradiography (right panel). (B) HflX(C) was treated with [α‐^32^P] GTP followed by UV crosslinking (lanes 1) or no UV treatment (lanes 2). The gel was stained by Coomassie Blue (left panel) and also subjected to autoradiography (right panel). Lane M represents molecular weight markers. (C) GTPase activity of HflX(C). The time kinetics of liberation of phosphate from GTP by HflX(C) is shown. Data plotted were obtained from three independent experiments.

We assayed for the GTP‐binding ability of HflX, HflX(C), and HflX(N) using [α‐^32^P] GTP and UV cross‐linking, as described under Materials and Methods. Like HflX, HflX(C) also exhibited strong GTP binding, but GTP binding for HflX(N) was weak (Fig. 3A). GTP binding for HflX(C) was also tested in the absence of crosslinking. No band appeared in the corresponding autoradiogram (Fig. 3B). Moreover, HflX(C) also released inorganic phosphate from GTP (Fig. 3C), exhibiting a GTPase activity akin to HflX.

#### Characterization of the HflX(C) phosphorylation reaction

**Figure 4:**
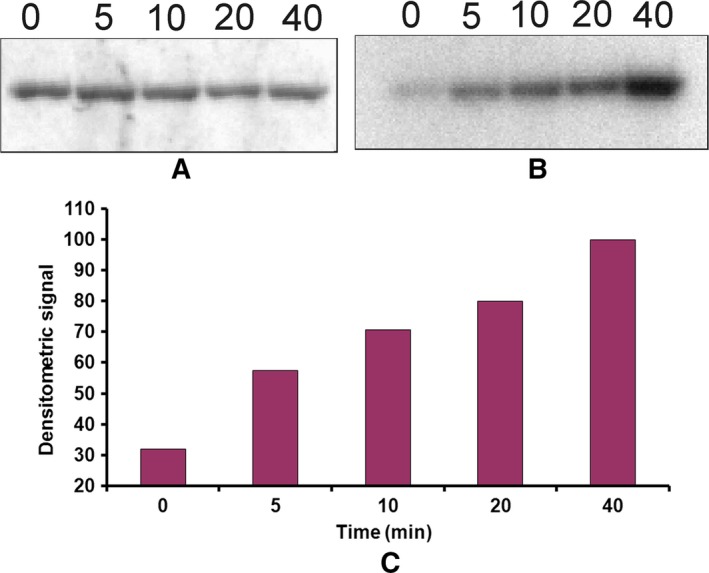
Time course of autophosphorylation of HflX(C). The autophosphorylation reaction was carried out for varying time intervals as indicated, using 5 μm of protein and 10 μCi [γ^32^‐P] GTP, followed by 15% SDS/PAGE. Both Coomassie blue staining (A) and autoradiography (B) of the same gel are shown, along with a column chart for the extent of autophosphorylation as a function of time (C), obtained from densitometry of the autoradiogram.

Autophosphorylation of HflX(C) was assayed in the presence of [γ^32^‐P] GTP. Aliquots of 10 μL were removed at different time points (0–40 min) and electrophoresed on a 15% SDS/PAGE. The gel was stained with Coomassie blue (Fig. 4A), de‐stained, dried, and autoradiographed (Fig. 4B). It is clear that the incubation of the protein with GTP led to autophosphorylation of HflX(C) that increased with time (Fig. 4C).

**Figure 5:**
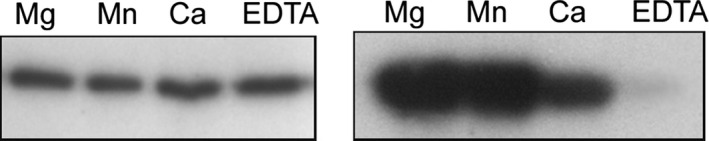
Autophosphorylation of HflX(C) in the presence of different divalent cations. Autophosphorylation of HflX(C) was carried out as stated earlier, for 40 min, in the presence of Mg^++^, Mn^++^, Ca^++^, or EDTA. Both the Coomassie blue stained gel (left panel) and the autoradiogram (right panel) are shown.

When the same autophosphorylation was carried out in the presence of different divalent cations (Mg^2+^, Mn^2+^, and Ca^2+^), HflX(C) showed a high degree of autophosphorylation for Mg^2+^ and Mn^2+^. The band intensities for these cations were comparable, and exceeded that for Ca^2+^ (Fig. 5). EDTA abolished the autophosphorylation completely, emphasizing the importance of divalent cations in autophosphorylation by HflX(C). It may be noted that a similar result was obtained for HflX, with the maximum band intensity being observed for Mn^2+^ 11.

#### Autophosphorylation of HflX(C) is intramolecular

**Figure 6:**
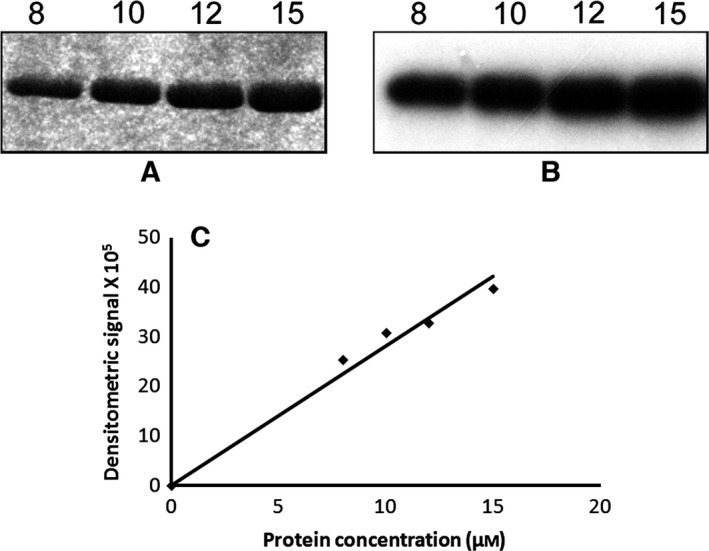
Autophosphorylation of HflX(C) increases linearly with protein concentration. Various indicated concentrations of HflX(C) (8–15 μm) were subjected to autophosphorylation followed by 15% SDS/PAGE and staining by Coomassie Blue (A) or autoradiography (B). About 10 μCi [γ^32^‐P] of GTP was used in each reaction. The bands shown in (B) were quantitated by densitometry and plotted against protein concentration (C).

The autophosphorylation reaction of HflX(C) carried out at different concentrations of the protein (8–15 μm) showed that the rate of the reaction was independent of HflX(C) concentration (Fig. 6). Such a first‐order kinetics with respect to concentration suggests an intramolecular phosphorylation mechanism 12.

#### Nature of the phosphorylated amino acid

Phosphorylated amino acid residues in proteins are commonly classified into three main groups: (a) *O*‐phosphates or *O*‐phosphomonoesters that are formed by phosphorylation of the hydroxyamino acids serine, threonine, and tyrosine; (b) *N*‐phosphates or phosphoramidates, produced by the phosphorylation of the basic amino acids arginine, histidine, and lysine; and (c) acylphosphates or phosphate anhydrides, generated by the phosphorylation of acidic amino acids (aspartic acid and glutamic acid). A fourth group, somewhat rare, includes S‐phosphates or thioesters of the sulfhydryl‐containing amino acid cysteine 19.

**Figure 7:**
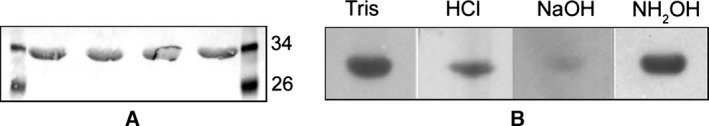
Chemical stability assay for autophosphorylated HflX(C). Equal amounts of HflX(C) (15 μm) were run in four individual lanes on a 15% SDS/PAGE followed by transfer to a polyvinylidene fluoride (PVDF) membrane and staining with Ponseau S (A). Molecular weight markers were run in the terminal lanes. Each of the protein lanes was incised and subjected to treatment with 50 mm Tris‐HCl (pH 7.5), 1 m HCl, 3 m NaOH, or 0.8 m NH_2_ OH at 42 °C followed by autoradiography (B).

To ascertain which type of phosphorylation is occurring for the autophosphorylation of HflX(C), chemical stability test for phospho‐amino acids was performed, as described in 19. Compared with the control (treated with the neutral buffer Tris‐HCl, pH 8), HflX(C) retained the radiolabel after treatment with HCl or hydroxylamine, but not when treated with NaOH. In the latter case, the level of the radiolabel retained was below 10% of that for the control lane (Fig. 7). Hence, the phosphate link in phosphorylated HflX(C) was an *O*‐phosphate linkage but not a phosphotyrosine, implicating the involvement of a serine or a threonine residue in the autophosphorylation reaction.

#### Identification of the autophosphorylation residue

**Figure 8:**
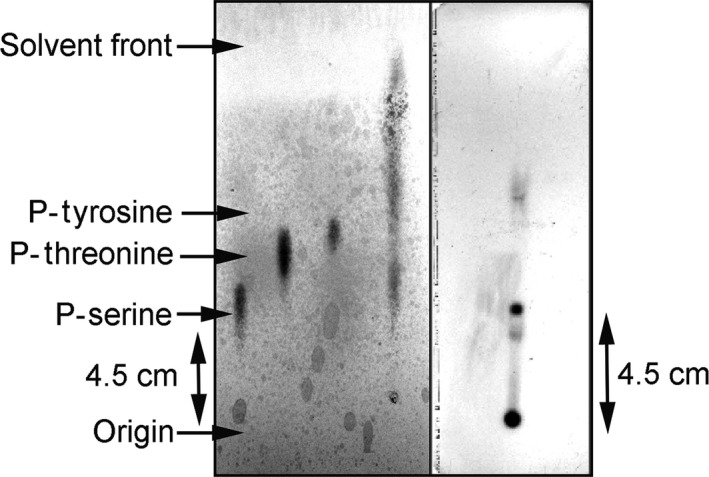
Identification of the residue of HflX(C) getting autophosphorylated, by thin layer chromatography. HflX(C) was subjected to autophosphorylation for 40 min as stated earlier, run on 15% SDS/PAGE, transferred to a polyvinylidene fluoride (PVDF) membrane and digested by 6N HCl at 110 °C for 60 min. The digested protein was spotted on a TLC plate and run with the appropriate buffer (lane 4). Control phosphoamino acids (P‐Tyr, P‐Thr and P‐Ser) were also run in neighboring lanes (1–3) along with the digested protein. The plate was stained by 0.1% ninhydrin (left panel). The same plate was also autoradiographed (right panel). Only the lane corresponding to the protein is shown.

Having narrowed the choice for the residue that is phosphorylated to Ser or Thr, we sought to identify this residue by chemical means according to 20. The protein was phosphorylated, digested by acid and spotted on to a thin layer cellulose plate and run with the TLC buffer along with control phosphoamino acids (i.e.*,* P‐Tyr, P‐Thr and P‐Ser), and also autoradiographed (Fig. 8). The single spot in the autoradiogram corresponded to the phosphoserine spot of the ninhydrin‐stained plate. Therefore, autophosphorylation by the protein is causing phosphorylation at a serine residue.

#### Sites of autophosphorylation in HflX

**Figure 9:**
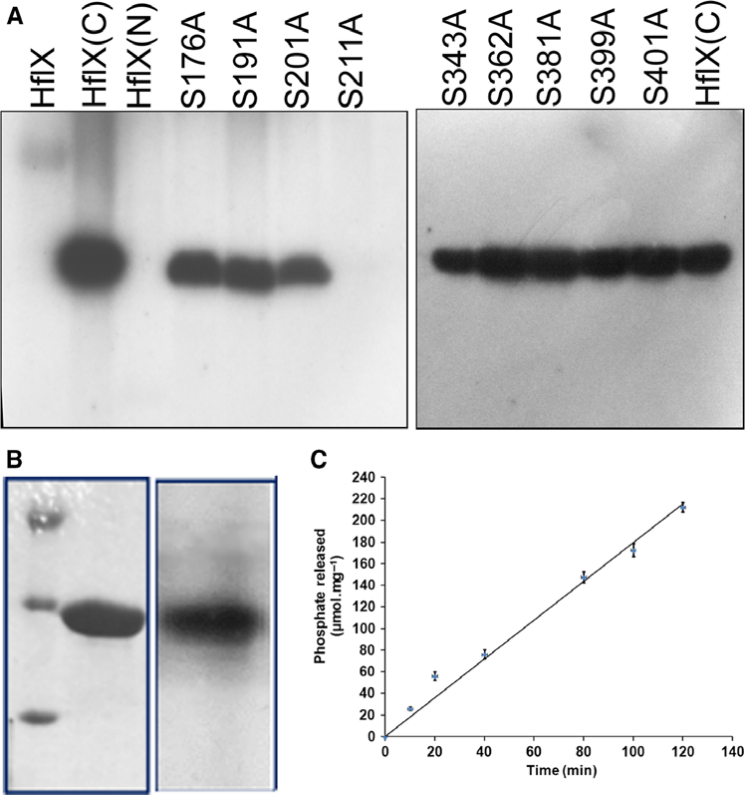
S211A mutation in HflX(C) leads to a loss of its autophosphorylation activity but not the GTP‐binding or GTPase activities. (A) Each of the nine Ser residues of HflX(C) was mutated to Ala and tested for autophosphorylation. Following the autophosphorylation reaction, the products were run on 15% SDS/PAGE followed by autoradiography. (B) GTP binding and (C) GTPase activity of S211A, assayed as in Fig. 3.

HflX(C) has nine serine residues. Each of these was mutated to alanine (S176A, S191A, S201A, S211A, S343A, S362A, S381A, S399A, S401A) and tested for autophosphorylation as before, by running the products on 15% SDS/PAGE followed by Coomassie Blue staining (not shown) and autoradiography. No phosphorylation occurred for the mutant S211A, unlike all other mutants (Fig. 9A). When this mutant was tested for GTP binding (Fig. 9B) or GTPase activity (Fig. 9C), it was found that the S211A protein could bind GTP and possessed GTPase activity. When the same mutation was effected on full‐length HflX, a similar result was obtained (not shown). Replacement of S211 by ala on HflX abolished the autophosphorylation activity of HflX, presumably due to the loss of the phosphorylation site, while its GTP binding and GTPase activities were retained.

## Discussion

The autophosphorylation activity of HflX 11, where GTP serves as a phosphate donor, emphasizes its similarities with other P‐loop GTPases. In general, GTP‐binding leads to the molecule being switched to an ‘activated’ form. Many such activated monomeric GTP‐binding proteins exhibit autophosphorylation. Similar GTP‐mediated autophosphorylation is also reported for other members of TRAFAC‐GTPases, such as the small protein Era from *E. coli* that is essential for bacterial growth, carbon metabolism, stringent response, and cell division 21, 22. Era is significantly autophosphorylated on either a serine or a threonine residue in the Switch I domain 12. Likewise, the GTPase Obg from *B. subtilis,* which is needed for growth and sporulation, is reported to be autophosphorylated, most likely at His189 13. Similarly, *C. crescentus* CgtA, a homolog of *B. subtilis* Obg, is essential for cell viability 23. Generally, autophosphorylation is a consequence of the juxtaposition of conserved amino acids to the γ‐phosphate of GTP. However, juxtaposed threonine residues (Thr192, Thr193) of CgtA to the γ‐phosphate of GTP remain unphosphorylated despite the protein being autophosphorylated at serine/threonine residues 14. Guanine nucleotide‐binding phosphoproteins have been recognized as components of signal trunduction pathways in eukaryotes 24, but the effect of autophosphorylation of guanine nucleotide binding phosphoproteins on cellular functions remains to be deciphered in prokaryotes. Nevertheless, a relationship between GTP‐dependent autophosphorylation of HflX using Mn as a primary cofactor and Mn homeostasis has been proposed 11. Autophosphorylation of HflX therefore merits a detailed study, toward which the present work is an essential first step.

It may be noted that full‐length HflX exhibited rather weak GTP binding, which was enhanced upon ribosome binding 25. The GTPase activity of the full‐length protein was also weak, and was significantly increased when the N‐terminal part of HflX was deleted 8, 15 (our unpublised work). In this paper we have shown that the autophosphorylation activity of HflX was also significantly enhanced when its N‐terminal part was absent. These results together suggest that the N‐terminal part of HflX masks its GTP binding/GTPase/autophosphorylation activities, which are correlated.

The results of this work demonstrate that autophosphorylation of HflX(C) was the result of non‐acyl covalent attachment of the gamma phosphate as seen by the ability of [γ‐^32^P]GTP to radiolabel HflX(C) and the stability of the label to boiling. Phosphorylation increased linearly with protein concentration of HflX(C), indicating that this phenomenon was intramolecular. This autophosphorylation also increased with time (Fig. 4), and divalent cations were essential for the autophosphorylation (Fig. 5).

From chemical stability studies, phosphorylated HflX(C) was found to be acid and hydroxylamine stable but base labile, suggesting that the phosphorylated amino acid(s) were either serine or threonine. Further, from thin layer chromatography it was found that the autophosphorylated residue is Serine.

The presence of Ser/Thr kinases in a broad range of microbial pathogens including Streptococcus, Mycobacteria, Yersinia, and Listeria, suggests that Ser/Thr phosphorylation is very common in prokaryotes 26. Thus, a large proportion of phosphoserine (phosphothreonine : phosphoserine = 1 : 38) has been detected in *E. coli* 27. On an average, phosphoserine is the phosphoamino acid most represented in proteins not only in *E. coli* but also in the majority of other bacterial species hitherto analyzed, both *in vivo* and *in vitro* 27. From mutational studies we identified that Ser211 was the site for autophosphorylation. Interestingly, while no autophosphorylation was observed for the S211A mutant of HflX(C), it retained GTP binding and GTPase activities. It appears that the P‐loop sequence can tolerate the change GX_4_GK(S/T) to GX_4_GKA. GTP binding followed by GTPase activity releases the gamma phosphate from GTP, which is covalently attached to the S211 residue in the P‐loop itself. We introduced the same S211A mutation into the full‐length protein and found that it had no autophosphorylation activity in the presence of γ‐[^32^P]GTP (data not shown), reinforcing the above conclusion.

The full‐length protein HflX displayed a low level of autophosphorylation in the presence of Mg^2+^. However, HflX(C) was found to be significantly autophosphorylated in the presence of both Mg^2+^ and Mn^2+^, while HflX(N) did not exhibit any autophosphorylation activity. This observation suggests that HflX(N) may have metal ion cofactor‐specific influence on the HflX(C) domain. Interestingly, similar crosstalk between HflX(N) and HflX(C) has been observed in GTP and ATP hydrolysis reactions, where each of the nucleotide‐binding domains influences the kinetics of NTP hydrolysis 15.

Why does HflX prefer Mn^2+^ for its autophosphorylation activity, while for HflX(C), both Mg^2+^ and Mn^2+^ are favored? Various bacterial GTPases that have autophosphorylation activity depend upon Mg^2+^, Mn^2+^, or other divalent cations in different ways. The autophosphorylation activity of Obg from *Mycobacterium tuberculosis* depends fully upon Mg^2+^ 28, while that for *E*. *coli* Era shows no preference for either Mg^2+^, Mn^2+^, or Ca^2+^ 12. On the other hand, autophosphorylation for Pkn2 from *Myxococcus xanthus* is supported by various divalent cations such as Mg^2+^, Mn^2+^, Co^2+^, or Zn^2+^ with a clear preference for Mn^2+^ 29. The fact that the ion specificity for HflX shifts upon deletion of its N‐terminal part indicates a subtle but significant change in the conformation of the molecule that retains the autophosphorylation activity but with an altered ion specificity. This hypothesis is supported by the enhancement of autophosphorylation activity in HflX(C) compared to HflX.

## Author contributions

DD, KB, AG and PP planned and designed the project, AG and KB obtained the data, AG, DD and PP wrote the paper.

## Supporting information


**Table S1.** Sequences of primers used in this study.

**Table S2.** Compositions of various buffers used in this work.
