# Supplementary material for: Characterization of the autophosphorylation property of HflX, a ribosome‐binding GTPase from Escherichia coli
Source: FEBS Open Bio. 2016 Jun 8;6(7):651–9. doi: 10.1002/2211-5463.12065 (PMC4932445; doi:10.1002/2211-5463.12065)
Supplement: Supplementary file 1 — Table S1. Sequences of primers used in this study. [file FEB4-6-651-s001.docx]

**Table S1: Sequences of primers used in this study.**

| **Primers** | **Sequence (5’-3’)** |
| --- | --- |
| XCF | ATG GGT CGC GGA TCC TTG TTT GAC CGT TAT G |
| XCR | CTC CGT CGA CAA GCT TTT AGA TCA GGT AAT CG |
| XNF | ATG GGT CGC GGA TCC TTG TTT GAC CGT TAT G |
| XNR | CTC CGT CGA CAA GCT TGC GAT TAC GCA ACA AA |
| S211AF | AAC GCC GGT AAA GCT ACC CTT TTC AAT |
| S211AR | ATT GAA AAG GGT AGC TTT ACC GGC GTT |
| S343AF | CGT GTC TGG CTT GCC GCA CAG ACC GGA |
| S343AR | TCC GGT CTG TGC GGC AAG CCA GAC ACG |
| S362AF | ACG GAG CGG CTT GCC GGC GAG GTGGCG |
| S362AR | CGC CAC CTC GCC GGC AAG CCG CTC CGT |
| S381AF | CGC CAC CTC GCC GGC AAG CCG CTC CGT |
| S381AR | CTG ATA AAA ACG AGC TCT CAG ACG CCC |
| S399AF | GAG GAG GAC GGC GCC GTA AGT CTG CAA |
| S399AR | TTG CAG ACT TAC GGC GCC GTC CTC CTC |
| S401AF | GAC GGC AGC GTA GCT CTG CAA GTT CGT |
| S401AR | ACG AAC TTG CAG AGC TAC GCT GCC GTC |
