# Supplementary material for: Characterization of the autophosphorylation property of HflX, a ribosome‐binding GTPase from Escherichia coli
Source: FEBS Open Bio. 2016 Jun 8;6(7):651–9. doi: 10.1002/2211-5463.12065 (PMC4932445; doi:10.1002/2211-5463.12065)
Supplement: Supplementary file 2 — Table S2. Compositions of various buffers used in this work. [file FEB4-6-651-s002.docx]

**Table S2: Compositions of various buffers used in this work.**

| **Buffer** | **Composition** |
| --- | --- |
| Buffer A | 20mM Tris-Cl (pH 8), 500 mM NaCl, 10% glycerol, 15 mM Imidazole (pH 8) and 100 μg/ ml PMSF |
| Buffer L | 20 mM Tris-Cl (pH 8), 500 mM NaCl, 8 M urea, 15 mM imidazole (pH 8) and 100 μg /ml PMSF |
| Buffer DU4 | 20 mM TrisCl (pH 8), 200 mM NaCl, 4 M urea |
| Buffer DU2 | 20 mM TrisCl (pH 8), 200 mM NaCl, 2 M urea |
| Buffer DU0 | 20 mM TrisCl (pH 8), 200 mM NaCl |
| Buffer DGE | 20mM TrisCl (pH 8), 200 mM NaCl, 5% glycerol, 2 mM EDTA |
| Buffer P | 20 mM Tris-HCl, 200 mM NaCl, 5 mM MgCl_2_, 1 mM PMSF, 1 mM DTT |
| Buffer G1 | 20 mM Tris-HCl, 200 mM NaCl, 5 mM MgCl_2_, 1 mM DTT |
| Buffer G2 | 20 mM Tris-HCl, pH 8; 200 mM NaCl, and 2 mM DTT |
| Buffer T | CH_3_CH_2_COOH, 1M NH_4_OH and CH_3_CH(OH)CH_3_ = 45:17.5:17.5, V/V |
